# The associations between environmental quality and preterm birth in the United States, 2000–2005: a cross-sectional analysis

**DOI:** 10.1186/s12940-015-0038-3

**Published:** 2015-06-09

**Authors:** Kristen M. Rappazzo, Lynne C. Messer, Jyotsna S. Jagai, Christine L. Gray, Shannon C. Grabich, Danelle T. Lobdell

**Affiliations:** Oak Ridge Institute for Science and Education at the U.S. Environmental Protection Agency, National Center for Environmental Assessment, U.S. Environmental Protection Agency, Research Triangle Park, NC USA; School of Community Health; College of Urban and Public Affairs, Portland State University, Portland, OR USA; Division of Environmental and Occupational Health Sciences, School of Public Health, University of Illinois, Chicago, Chicago, IL USA; Gillings School of Global Public Health, University of North Carolina, Chapel Hill, NC USA; Oak Ridge Institute for Science and Education at the U.S. Environmental Protection Agency, National Health and Environmental Effects Research Laboratory, U.S. Environmental Protection Agency, Chapel Hill, NC USA; National Health and Environmental Effects Research Laboratory, U.S. Environmental Protection Agency, Chapel Hill, NC USA

**Keywords:** Environmental quality, Air quality, Water quality, Land quality, Built environment, Sociodemographic, preterm birth

## Abstract

**Background:**

Many environmental factors have been independently associated with preterm birth (PTB). However, exposure is not isolated to a single environmental factor, but rather to many positive and negative factors that co-occur. The environmental quality index (EQI), a measure of cumulative environmental exposure across all US counties from 2000—2005, was used to investigate associations between ambient environment and PTB.

**Methods:**

With 2000–2005 birth data from the National Center for Health Statistics for the United States (*n* = 24,483,348), we estimated the association between increasing quintiles of the EQI and county-level and individual-level PTB; we also considered environmental domain-specific (air, water, land, sociodemographic and built environment) and urban–rural stratifications.

**Results:**

Effect estimates for the relationship between environmental quality and PTB varied by domain and by urban–rural strata but were consistent across county- and individual-level analyses. The county-level prevalence difference (PD (95 % confidence interval) for the non-stratified EQI comparing the highest quintile (poorest environmental quality) to the lowest quintile (best environmental quality) was −0.0166 (−0.0198, −0.0134). The air and sociodemographic domains had the strongest associations with PTB; PDs were 0.0196 (0.0162, 0.0229) and −0.0262 (−0.0300, −0.0224) for the air and sociodemographic domain indices, respectively. Within the most urban strata, the PD for the sociodemographic domain index was 0.0256 (0.0205, 0.0307). Odds ratios (OR) for the individual-level analysis were congruent with PDs.

**Conclusion:**

We observed both strong positive and negative associations between measures of broad environmental quality and preterm birth. Associations differed by rural–urban stratum and by the five environmental domains. Our study demonstrates the use of a large scale composite environment exposure metric with preterm birth, an important indicator of population health and shows potential for future research.

## Background

Preterm birth (PTB), defined as live birth occurring before 37 weeks of completed gestation, is a marker for fetal underdevelopment and a risk factor for poor health outcomes in both the short and long term, including: infant mortality, neurodevelopmental problems, and growth issues [1–4]. The United States (US) has a PTB/live birth rate of 12 %, ranking the 54th highest out of 184 counties with known preterm birth rates [5]. The reduction of PTB is a national objective established by the Department of Health and Human Services and monitored through its Healthy People 2020 initiative [6]. As such, PTB can be used as an indicator of population or national health [7].

While individual characteristics such as maternal age, parity, smoking status, and maternal educational status are well-established risk factors [1], the causal mechanisms for PTB remain unclear and are thought to be multi-faceted. In recent years, the literature investigating environmental factors across several environmental domains, including the air, water, land, built, and sociodemographic, and PTB has grown considerably. This is particularly true in the area of air pollution research, where PTB has been associated with many contaminantes including particulate matter, ozone, and nitrogen dioxide [8–11]. PTB has also been associated with disinfection byproducts and atrazine in water, as well as exposure to other pesticides through agriculture [12–15]. In the non-chemical environment, PTB has been associated with neighborhood and socio-demographic characteristics, such as neighborhood-level unemployment status, income level, poor housing, or racial isolation [16, 17].

Though PTB is associated with many environmental factors, approaches that address the cumulative effect of multiple exposures simultaneously are not common. The typical approach is to examine single exposures independently. Studies may control for one or two co-pollutants within the media of interest, for example adjusting for ozone when examining particulate matter, but are unlikely to adjust for a broader set environmental factors or factors outside the media of interest, such as adjusting for air pollutants when examining water contaminants. Source apportionment and emissions-based methods are useful for examining “upstream” environmental exposures that encompass individual pollutants; however not all exposures in the broad environment lend themselves to these methods.

Indices that reduce multiple variables to a single representative measure are used as exposure variables in select environmental research. These methods have more commonly been used in built and social environment research, where examination of multiple variables describing neighborhood deprivation is a more useful metric of exposure than any one indicator used singularly [18–21]. Air pollution studies have also employed index methods to examine complex air mixtures [11]. However, index methods are typically used within a single domain of the environment (e.g., built or air) but not across environmental domains.

To deal with the multiplicity of simultaneous environmental exposures, the Environmental Quality Index (EQI) was constructed to represent multiple domains comprising the ambient environment [22]. By including information on air contaminants, water quality, agriculture, pesticide use, road density, housing, businesses, socioeconomics, crime, and other variables, the EQI represents exposures across the air, water, land, built environment, and sociodemographic environmental domains at the county level across the United States [22, 23]. The EQI consists of both a single unified county-level index (the overall EQI) and indices from each environmental domain, wherein associations with one domain may be examined while controlling for the other ambient environment to which an individual is exposed. Using the EQI, researchers are able to examine a more cumulative measure of the environment in a way that incorporates non-emitted exposures and does not require high statistical power.

In this cross-sectional study we use the EQI to investigate associations between PTB and simultaneous environmental exposures. We examine county-level prevalence of PTB in association with the EQI, as the EQI may reflect larger-scale exposure more appropriately than small scale or variable exposures, and to explore if there are drivers of county-level birth outcomes. We also examine individual-level odds of PTB in association with the EQI to understand the environmental contribution to PTB above and beyond individual-level covariates, and to investigate the ecological environmental exposures association with PTB at a level comparable to much of the previous environmental-PTB literature. Because the overall environment, as represented by the EQI, is comprised of measures that may influence health in a negative, neutral, or positive manner, and we wish to better understand the complex relationships between environmental quality and health, we also examine associations between domain indices. As environments differ across urban–rural status and different aspects may drive environmental quality in rural versus urban locations [24], we also investigated the associations between PTB and the EQI and domain indices stratified by rural–urban status.

## Methods

### Study population

The source population included live births provided by the National Center for Health Statistics (NCHS) for the entire United States for the years 2000–2005 (*n* = 24,483,348). The National Vital Statistics System of the NCHS is a repository of all vital records collected under state laws and compiled under Federal law; birth data available represent all births registered in the 50 States, the District of Columbia, and New York City [25]. The study population was restricted to singleton, non-anomalous births, with county identifiers, recorded gestational age, and residence within the same state as birth occurrence (*n* = 22,705,068). Two analytic sets were generated from the study population. The individual-level analytic set consisted of all births with complete covariate information (*n* = 22,156,095). For the county-level analysis, total births and PTBs were aggregated from the study population set for each county and prevalence of PTBs/total births were constructed (*n* = 3141 counties). Counties with fewer than 10 births or with no PTBs over the study period were excluded (*n* = 10; 2 from less urbanized counties, 8 from rural/isolated counties).

### Preterm birth

In both the county- and individual-level analyses, PTB was defined as a birth occurring between 20 and 36 weeks completed gestation (inclusive), with 37 completed weeks and above considered term birth. Gestational age was taken from the provided NCHS variable; this variable combines multiple methods of gestational estimation depending on available data. First, dates of birth and last normal menses was used to compute gestational age; if last normal menses was unavailable, then last menstrual period date was used to impute gestational age; if last menstrual period date was unavailable, then clinical estimate of gestation was used which may include ultrasound dating or be a clinicians estimate of gestational age at time of birth [26, 27]. If none of this information was provided on birth records the variable was marked as unknown due to insufficient data [26, 27].

### EQI

**Fig. 1:**
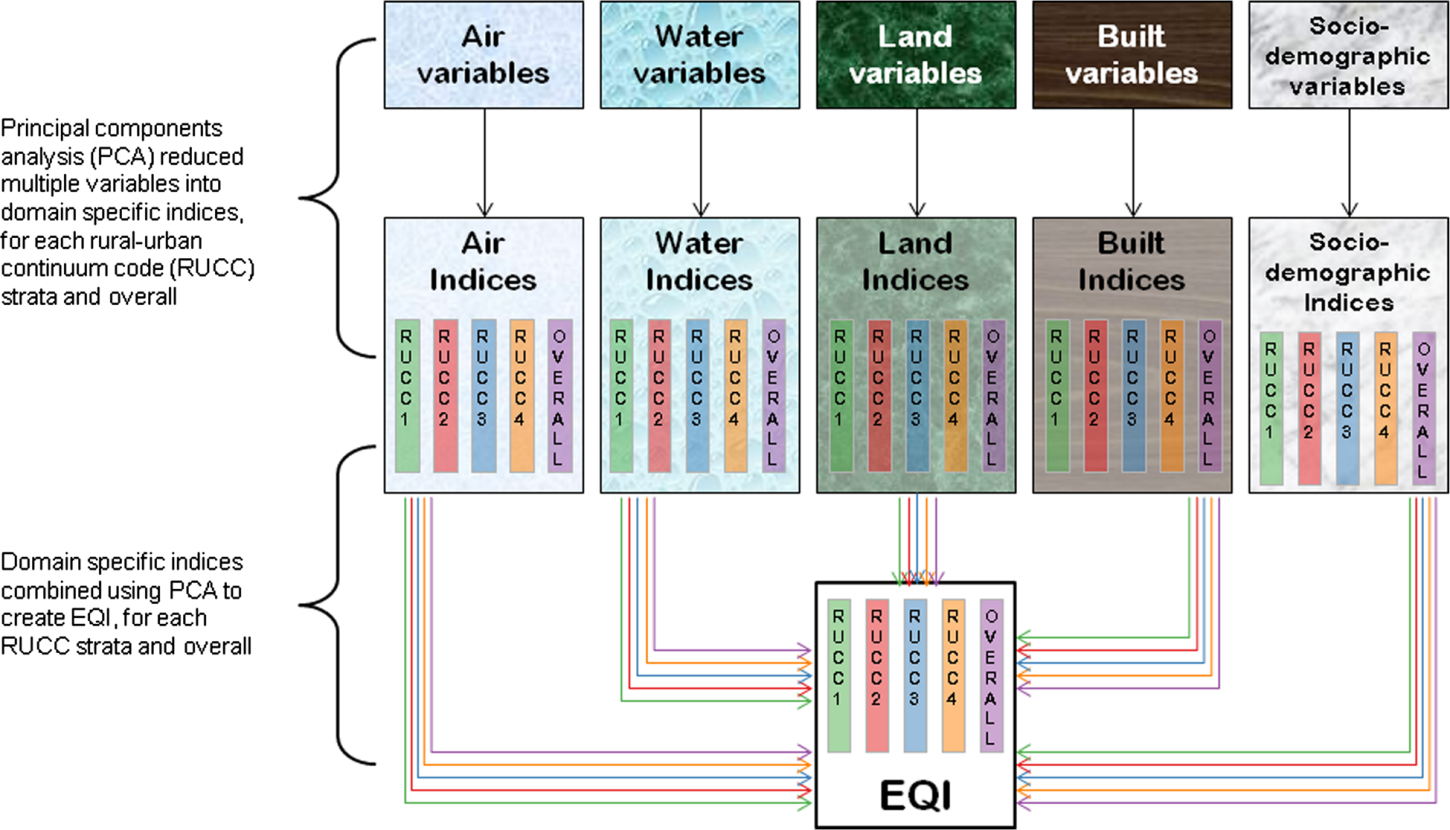
Principal components analysis concept for Environmental Quality Index (*EQI*). Performed for all counties and each of the four strata of the rural–urban continuum (*RUCC*) codes [22]

We used the EQI as an indicator of environmental quality at the county-level for the United States for the period of 2000 to 2005. Full methods of the EQI’s construction are described in Messer et al. [22], while data description can be found in Lobdell et al. [23]. Briefly, the EQI includes variables representing five environmental domains (air (*n* = 87), water (*n* = 80), land (*n* = 26), built (*n* = 14), and sociodemographic (*n* = 12)). Domain-specific variables were included in principal components analysis (PCA) and the first component was retained as that domain’s index (air index, water index, etc.). These indices were then combined, again using PCA, and the first component of this PCA was retained as the EQI. To account for variation in geography and environmental drivers, we repeated this process in four rural–urban strata: metropolitan-urbanized, non-metropolitan urbanized, less urbanized, and rural/isolated. These strata were collapsed from the nine rural–urban continuum codes (RUCC) defined by the USDA [28], as has been done previously [29, 30]. The result was six non-stratified indices (one overall EQI and five domain-specific EQIs) and six corresponding indices for each of the four RUCC strata (Fig. 1). Higher values for each index were constructed to correspond to poorer environmental quality.

### Data analyses–county-level

For the county-level analysis, we defined the county prevalence of PTB as the ratio of PTBs to all live births per county. The exposure variables for the analyses were: non-stratified and RUCC-stratified EQI, and non-stratified and RUCC-stratified domain-specific indices. Index values were linked to PTB prevalences by maternal county of residence. Linear regression was used to estimate prevalence differences (PDs) and 95 % confidence intervals (CIs) for upper 4 quintiles of each index compared to the lowest quintile; each domain quintile was entered into the model as an indicator value, which allows for non-monotonic associations with increasing values. Though indices were initially continuous, quantiles were more meaningful than the continuous variable as associations can be interpreted as the difference between the areas with the best environmental quality (lowest quintile category) and areas with poorer environmental quality. PDs were reported as proportions; positive PDs indicate an increase in PTBs, while negative PDs indicate a decrease in PTBs, with a null value at zero. Domain indices were entered simultaneously into models (i.e., air index results are adjusted for water, land, built and sociodemographic exposure). Due to the “upstream” (i.e., higher level and affected by fewer factors) and inclusive nature of the exposure, we identified few potential confounders for inclusion in the county-level models. However racial composition of counties may be a driver of environmental quality, due to siting of disamenities, environmental justice issues, as well as other issues [31]. Therefore, county-level proportion of minority residents (from census data) was included as a covariate.

### Data analysis–individual-level

For the individual-level analysis, the outcome was individual PTB status. The exposure variables for the analyses were: non-stratified and RUCC-stratified EQI, and non-stratified and RUCC-stratified domain-specific indices for maternal county of residence. We used multilevel logistic models accounting for clustering at the county-level to estimate odds ratios (ORs) and CIs for upper 4 quintiles of indices compared to the lowest quintile. In this analysis, the null value was one. Domain indices were entered simultaneously into models. We identified potential confounders a priori based on previous environmental literature and knowledge of factors influencing PTB. Models were adjusted for maternal age (<20, 20–29, 30–39, >39), education (less than high school, high school graduate, greater than high school), and marital status (married, unmarried). These maternal demographic factors are risk factors of PTB and associated with socio-economic status, which can influence where a woman resides and therefore environmental exposures. We adjusted for individual level education (as a covariate), even though area-level education is included in the sociodemographic domain, to enable us to estimate the area-level effects on health outcomes, over and above the individual-level characteristics. Further, both characteristics may act separately on health outcomes [32]. All adjustment variables were extracted from birth certificates.

### Sensitivity analyses

We conducted two sensitivity analyses in this study. In the first, we examined joint effect estimates with simultaneous quintile increases in all domain indices. We did this because a simultaneous change from 1st to 5th quintile in all five indices is not the same as a 1st to 5th quintile change in the overall index. By examining overall trends we can investigate the sensitivity of the estimated effects from the EQI. Secondly, because access to prenatal care for uninsured women varied at the state level and this access may influence risk of PTB, we also performed a sensitivity analysis with adjustment for state as a proxy for available care.

## Results

### Population description

There were 3131 counties included in the analysis. Of these, 35 % (1089) were metropolitan-urbanized, 10 % (323) were non-metropolitan urbanized, 34 % (1057) were less-urbanized, and 21 % (662) were rural/isolated. County-level prevalence of PTB had a mean (standard deviation) of 10.42 % (4.12 %), with an interquartile range of 4.15 %.

There were 22,609,391 women included in the individual-level analysis (Table 1). Births primarily occurred in the most metropolitan areas (84 % overall). Women with preterm births were more likely to be Black, unmarried, and have attained lower educational status than women with term births. Women with preterm births were also more likely to be at either end of the age distribution (<20 or >39) than women with term births. The births that were excluded because of missing covariate information were similar in their preterm birth proportion to the births retained for the analysis; excluded births had a PTB proportion of 11 %, while included births had a PTB proportion of 10 %.

**Table 1:** Demographics of individuals in study population by preterm status (*n* (%))

Characteristic	Preterm	Term
Total	2,335,880 (10)	20,369,188 (90)
Maternal race		
White, non-Hispanic	1,139,435 (49)	11,719,095 (58)
Black, non-Hispanic	524,453 (22)	2,771,770 (14)
Hispanic	524,477 (22)	4,462,084 (22)
Other	128,440 (5)	1,239,083 (6)
Missing	19,075 (1)	177,156 (1)
Marital status		
Married	1,308,539 (56)	13,482,654 (66)
Unmarried	1,027,341 (44)	6,886,534 (34)
Maternal age		
< 20	335,318 (14)	2,213,480 (11)
20–29	1,186,750 (51)	10,700,735 (53)
30–39	744,495 (32)	6,982,810 (34)
> 39	69,317 (3)	472,163 (2)
Maternal education		
< HS	613,828 (26)	4,295,592 (21)
HS	761,100 (33)	6,145,251 (30)
> HS	923,370 (40)	9,665,601 (47)
Missing	37,582 (2)	262,744 (1)
Rural–urban stratum		
Metropolitan urbanized	1,956,542 (84)	17,214,809 (85)
Nonmetropolitan urbanized	156,155 (7)	1,320,384 (6)
Less urbanized	179,568 (8)	1,469,263 (7)
Thinly populated/rural	33,138 (1)	279,532 (1)

### County-level EQI results

**Fig. 2:**
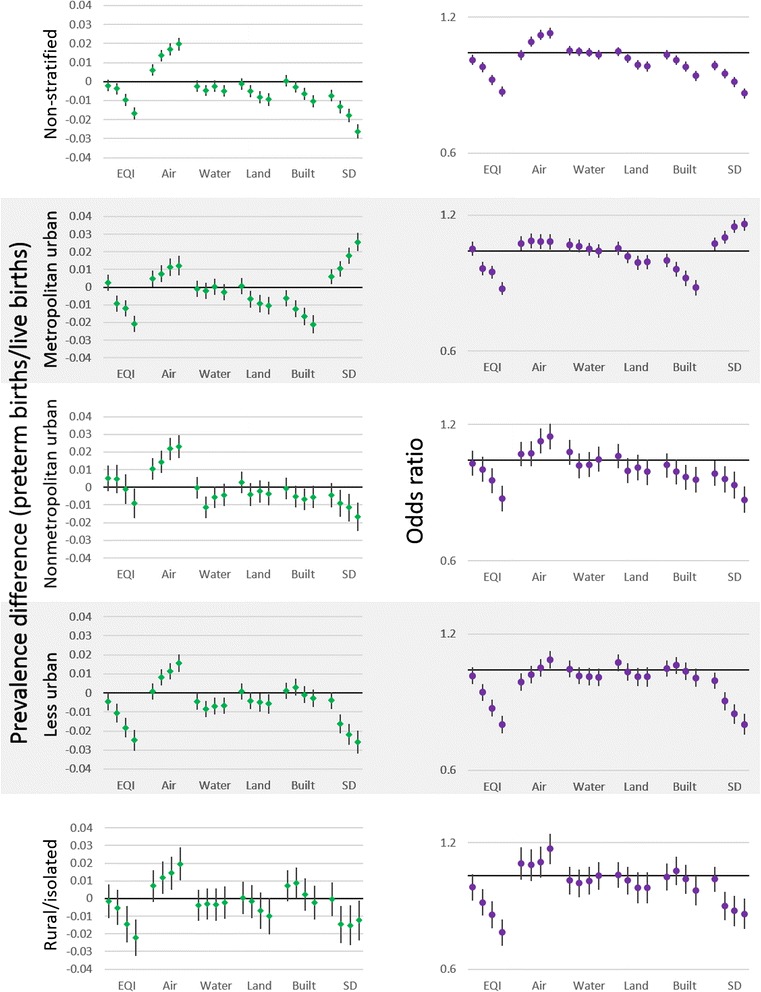
Prevalence differences for county (*left*) and odds ratios for individual (*right*) analyses of preterm birth and environmental quality across rural–urban strata. Quintiles compared to lowest (“best environmental quality”) increasing from left to right. County-level analyses adjusted for race (proportion of minority residents), individual-level analyses adjusted for maternal race, education, age at delivery. Analyses of domain specific indices were adjusted for other domain indices (air for water, land, built, and sociodemographic). SD = Sociodemographic

Models of county-level PDs were used to explore drivers of county-level PTB. Associations between the air domain index and PTB were generally positive for county-level analyses; with worsening air quality prevalence of preterm birth increased (Fig. 2). For example, in the non-stratified county level analysis the PD (95 % CI) for highest quartile compared to the lowest was 0.0196 (0.0162, 0.0229). Patterns of associations between the air domain and PTB were also similar across rural–urban strata, in that there was an increase in PTB prevalence with worsening air quality. However, the magnitude of the increase differed across strata, with the metropolitan-urbanized strata having the lower PDs, and in the more urban strata there was the appearance of a plateauing of effect estimate at the 4th and 5th quintiles.

Effect estimates with the water domain index tended to be negative and near-null, or null. For example, the PD for the 4th quintile compared to the 1st quintile in the less urbanized stratum was −0.0069 (−0.0112, −0.0025) and for the metropolitan urbanized stratum was 0.0003 (−0.0043, 0.0048). Patterns of PDs appeared to be mostly flat, with all estimates being negative or null rather than trending in a particular direction. We observed consistent negative effect estimates only in the less urbanized counties, while PDs in the other strata either remain null or fluctuate around the null.

Associations for the land and built domains were similar and both tended to be either negative or null, with generally decreasing PTB as built or land quality worsened. For example, in the land domain (non-stratified) the PD for the 3rd quintile was −0.0051 (−0.0082, −0.002), and the PD for the 5th quintile was −0.0094 (−0.0127, −0.0061). The most consistent effect estimates were in the metropolitan urbanized strata, where a negative trend was observed. Other strata also showed negative PDs, however estimates were similar with worsening quality.

Effect estimates for the sociodemographic domain had clear trends in all strata; however the direction of those trends changed depending on the particular rural–urban stratum. For example, in the metropolitan urbanized stratum (highest to lowest quintile) the PD was 0.0256 (0.0205, 0.0307), while in the less urbanized stratum the PD was −0.0258 (−0.0318, −0.0199). PDs in the nonmetropolitan urbanized, less urbanized, and rural/isolated strata all showed decreasing ORs with worsening sociodemographic quality. However, in the metropolitan urbanized strata the opposite trend was shown, with worsening sociodemographic quality associated with increasing prevalence of PTB.

The EQI, which combines all individual domains into a single index, showed consistent decreases in PTB with increasing quartiles or worsening environmental quality. PDs for the non-stratified 2nd to 5th quintiles were: −0.0021 (−0.0052, 0.001), −0.0038 (−0.007, −0.0007), −0.0098 (−0.013, −0.0066), −0.0166 (−0.0198, −0.0134).

### Individual-level EQI results

To understand the environmental contribution to PTB above and beyond individual-level covariates we examined individual-level ORs. As in the county-level analysis, associations between the air domain and PTB were generally positive in the individual-level analysis (Fig. 2). However, trends were not as straightforward. Non-stratified ORs showed increases, but plateau between the 4th and 5th quintiles. In the metropolitan urbanized stratum, ORs were elevated from the null, but were also flat across quintiles. While in the nonmetropolitan urbanized and less urbanized strata there was a more clear increasing trend (e.g., nonmetropolitan urbanized, 3rd quintile OR: 1.04 (0.97, 1.10), 5th quintile OR: 1.13 (1.06, 1.21)). In the rural/isolated stratum ORs were elevated from the null but increase from one another only from the 4th to 5th quintiles (OR 4th quintile: 1.08 (0.99, 1.17), OR 5th quintile: 1.16 (1.07, 1.26)).

In the water domain, ORs were generally null, with the exception of the less urbanized stratum where they were slightly negative. Similarly, ORs for the land domain were near-null or negative; non-stratified ORs showed a negative trend (OR 2nd quintile: 1.01 (0.99, 1.04), OR 5th quintile: 0.99 (0.97, 1.01)), while the trend was not clear in the stratified results.

Associations for the built environment and PTB were generally negative. In the metropolitan urban and nonmetropolitan urban strata, and in the non-stratified analysis, there were negative trends, with worsening built environment being associated with lower ORs of PTB (5th quintile: 0.89 (0.87, 0.91)). In the less urban and rural/isolated strata the lower quintiles had null associations with PTB, while the highest quintile had an inverse OR (rural/isolated OR, 2nd quintile: 1.00 (0.92, 1.07), 5th quintile: 0.92 (0.85, 1.00)).

In the sociodemographic domain, patterns of ORs were similar to PDs in the individual-level analysis; ORs had clear trends in all strata, but the trend direction was different in the metropolitan urban stratum. In the non-stratified, nonmetropolitan urban, less urban, and rural/isolated strata the ORs for PTB decreased with worsening sociodemographic environmental quality (increasing quintiles) (ex ORs for non-stratified analysis 2nd quintile: 0.94 (0.92, 0.96), 5th quintile: 0.81 (0.79, 0.83)). While the metropolitan urban stratum had the opposite trend, with worsening sociodemographic quality associated with increasing ORs for PTB (2nd quintile: 1.04 (1.00, 1.07), 5th quintile: 1.15 (1.11, 1.18)).

With worsening overall environmental quality/increasing quartiles of the EQI, ORs for PTB were negative and consistently decreased. ORs for the non-stratified 2nd to 5th quintiles: 0.96 (0.94, 0.99), 0.93 (0.91, 0.95), 0.87 (0.85, 0.89), 0.82 (0.80, 0.84).

### Sensitivity analyses

Joint effect estimates with all domains increasing simultaneously were similar to effect estimates using the EQI as exposure, though shifted upwards in the metropolitan urban strata; overall trends were the same between analyses (Table 2). Effect estimates from analyses with state-level adjustment were similar to those without state-level adjustment (data not shown).

**Table 2:** Effect estimates for simultaneous joint-change sensitivity analyses

		County-level prevalence differences		Individual-level odds ratios	
Strata		EQI	Joint-change	EQI	Joint-change
Overall	Q1	ref	ref	ref	ref
	Q2	−0.0021 (−0.0052, 0.001)	−0.0047 (−0.0112, 0.0018)	0.96 (0.94, 0.99)	0.93 (0.89, 0.99)
	Q3	−0.0038 (−0.007, −0.0007)	−0.0123 (−0.0186, −0.0059)	0.93 (0.91, 0.95)	0.90 (0.85, 0.94)
	Q4	−0.0098 (−0.013, −0.0066)	−0.0178 (−0.0241, −0.0115)	0.87 (0.85, 0.89)	0.83 (0.79, 0.87)
	Q5	−0.0166 (−0.0198, −0.0134)	−0.0312 (−0.0365, −0.0259)	0.82 (0.80, 0.84)	0.74 (0.71, 0.77)
Metropolitan urbanized	Q1	ref	ref	ref	ref
	Q2	0.0025 (−0.002, 0.0071)	0.0042 (−0.0052, 0.0135)	1.01 (0.98, 1.05)	1.07 (1.00, 1.15)
	Q3	−0.0092 (−0.0138, −0.0046)	−0.003 (−0.0122, 0.0062)	0.92 (0.88, 0.95)	1.02 (.95, 1.1)
	Q4	−0.012 (−0.0166, −0.0075)	0.0033 (−0.0059, 0.0126)	0.90 (0.87, 0.93)	0.98 (0.91, 1.05)
	Q5	−0.0207 (−0.0253, −0.0161)	0.0033 (−0.0065, 0.0131)	0.83 (0.80, 0.85)	0.94 (0.87, 1.01)
Non-metropolitan urbanized	Q1	ref	ref	ref	ref
	Q2	0.0049 (−0.0023, 0.0122)	0.0078 (−0.006, 0.0217)	0.99 (0.93, 1.05)	1.00 (0.88, 1.15)
	Q3	0.0046 (−0.0035, 0.0126)	−0.0154 (−0.0307, 0)	0.96 (0.90, 1.02)	0.82 (0.71, 0.94)
	Q4	−0.001 (−0.0094, 0.0074)	−0.0044 (−0.0183, 0.0095)	0.90 (0.85, 0.96)	0.84 (0.74, 0.95)
	Q5	−0.0091 (−0.0176, −0.0005)	−0.0069 (−0.0215, 0.0077)	0.82 (0.77, 0.88)	0.79 (0.70, 0.90)
Less urban	Q1	ref	ref	ref	ref
	Q2	−0.0046 (−0.0093, 0.0002)	−0.0059 (−0.0152, 0.0034)	0.97 (0.93, 1.01)	0.93 (0.85, 1.02)
	Q3	−0.0105 (−0.0155, −0.0055)	−0.0179 (−0.0273, −0.0085)	0.89 (0.85, 0.93)	0.82 (0.75, 0.90)
	Q4	−0.0183 (−0.0235, −0.013)	−0.0231 (−0.0329, −0.0134)	0.82 (0.79, 0.86)	0.75 (0.68, 0.82)
	Q5	−0.0249 (−0.0303, −0.0195)	−0.0253 (−0.0346, −0.0159)	0.76 (0.72, 0.79)	0.71 (0.65, 0.78)
Rural/isolated	Q1	ref	ref	ref	ref
	Q2	−0.0016 (−0.0112, 0.0081)	0.0104 (−0.0088, 0.0296)	0.94 (0.88, 1.01)	1.03 (0.88, 1.20)
	Q3	−0.0052 (−0.0151, 0.0048)	0.0012 (−0.0193, 0.0217)	0.87 (0.80, 0.93)	0.87 (0.73, 1.02)
	Q4	−0.0145 (−0.0248, −0.0043)	−0.009 (−0.0305, 0.0126)	0.81 (0.75, 0.87)	0.80 (0.67, 0.94)
	Q5	−0.0222 (−0.0326, −0.0117)	−0.0073 (−0.027, 0.0125)	0.73 (0.68, 0.79)	0.81 (0.70, 0.95)

## Discussion

We observed both strong positive and negative associations between measures of broad environmental quality and preterm birth. Associations differed by rural–urban stratum and by the five environmental domains. We observed associations in both county-level and individual-level analyses; those results tracked similarly, potentially indicating the EQI and domain specific indices as robust predictors of both county- and individual-level outcomes. Decreasing environmental quality (increasing EQI) was consistently associated with decreased county-level prevalence and individual-level odds of PTB; this is likely due to the combinations of the effects of individual domains. Worsening air quality (increasing air domain index) was consistently associated with increases in PTB, while the water, built, and land domain indices had null or negative associations with PTB. The sociodemographic domain index was positively associated with PTB in the metropolitan urbanized strata, but negatively associated with PTB in all other stratum and in the non-stratified analysis.

Few other studies have looked at composite environmental exposures in association with PTB. Of those that have, several examined sociodemographic or built environment indices, all based on similar techniques; these studies have primarily examined areas that would be classified as metropolitan urbanized [18–21]. Though there were variations in each index used, generally these studies found that higher deprivation/lower socioeconomic advantage was associated with poorer birth outcomes and increased PTB. We observed results similar to these studies when examining the most urban stratum (metropolitan urbanized). This may reflect the better quality of data or better defined “neighborhood” qualities for this stratum. There may also be different drivers of the sociodemographic environment in the less urban strata that were not adequately represented by the data available for the EQI.

Differences in patterns of association across rural–urban strata may be explained through differing contributions of variable loadings. In principal component analysis, variable loadings represent the strength of a variable’s contribution to the index value. In our analysis, these loadings differed across rural–urban strata; for example, in the sociodemographic domain index, percent of people at or below poverty level loaded highly positive in the metropolitan urban stratum (0.45) and highly negative for all other strata [22]. The different loadings across rural–urban strata may indicate differences in the environmental drivers of the observed associations across the strata, which potentially explains the observed variation in patterns of association, particularly in the sociodemographic domain. While variables were coded to load positively if they were associated with poor health outcomes, the tiered nature of the EQI construction may result in reversal of this coding if domain indices varied in opposite directions. It is also possible that uniformity or limited variation in the input variables to the EQI could lead to differential statistical power to detect associations across rural–urban strata. However, examining the ranges of variables, this generally does not seem to be a factor. For example, the ranges of percent earning greater than high school education across strata are: metropolitan urbanized 1.0–91.9; nonmetropolitan urbanized 1.9–92.1; less urban 0.7–84.8, and rural 0.4–85.4 [22]. Another possible explanation for observed differences is that the most accurate or complete data were often from the most urban counties. Therefore, it is possible that those counties were most representative of actual environmental quality exposure. In regard to the sociodemographic domain specifically, previous research has largely considered urban poverty when investigating poverty effects on health status. In the urban strata we see the results we might expect given previous work. Given the results in non-urban strata, it may be that rural poverty is substantially different than urban poverty and living in a non-urban area is not associated with the same risk that is observed in other studies because of their setting. This issue may be addressed in future versions of the EQI by attempting to identify better markers of the rural and suburban sociodemographic environment and incorporate those into the EQI.

For air pollution, Wilhelm et al. ([11]) examined associations with PTB using factor analysis from monitoring station measurements and land use regression estimates of pollutant concentration. In this study, the factors produced were not used as the main exposure variables, but used to identify the smallest number of pollutants that could be used as adjustment factors for co-pollutants. For example, rather than using the 1st factor as a stand in for the 13 pollutants with high loadings for that factor, ammonium nitrate PM_2.5_ was selected to represent all of them, and sensitivity analyses were performed by using a different variable and comparing results [11]. They found increased effect estimates with the representative variable for the 1st factor (representing: NO_2_, elemental carbon, organic carbon, diesel PM_2.5_, benzene, and PAHs, among others) but found null associations for other factors and negative effect estimates for ammonium sulfate PM_2.5_ [11]. There are a number of differences between our analyses and Wilhelm et al.’s; in particular, they used individual exposures while we employed ecologic exposures at the county level, and they used representative pollutants while we retain the composite metric. However, in both studies an increase of air pollution was associated with increases in PTB.

To our knowledge, no research has been performed that examines composite indices of water or land quality measures in association with PTB. Further, no research has examined cumulative environmental quality, including multiple environmental domains and both potentially beneficial and detrimental exposures in association with preterm birth.

In general, data were more readily available across all domains for the most urban areas, with the need for more reliance on estimation in the suburban and rural areas. The differential availability of data across strata gives rise to the possibility of differential misclassification based on urban–rural strata. Variables were chosen to represent domains as a whole, before stratification. It is possible that variable selection specific to a rural–urban stratum would result in different choices for each stratum. There are also likely to be differences in individual versus county exposure representation across strata. For example, in the less urban areas for the air domain, county level exposures were more likely to be reflective of personal exposure. We know that there was substantial heterogeneity in urban air concentrations [33–35] which was smoothed over with a county-level approach, but in areas where concentrations may not change as rapidly, a county-level approach may capture exposure as well as more granular measurements. As a corollary, individual exposures in areas where rapid change was expected may not be well represented, such as in highly urban areas. However, in our analysis county-level and individual-level assessment yielded similar results.

Other potential limitations include the broad nature of the exposure metric; the ecological nature of the EQI and the cross-sectional nature of this analysis mean that our exposure was distal to preterm birth. Mechanisms directly linking environmental quality to PTB were also not well described in the literature and unlikely to act through a single pathway. The EQI represents the environment over a 5-year period at the county level, and may mask variation in environmental factors over this period. While the individual domains of the EQI were constructed to be negative (e.g., higher concentrations of air pollution are a negative aspect of environmental quality), some factors may be either negative, positive, or neutral and this may obfuscate final interpretation of the domains to which these factors contribute. For example, high street density in the built environment domain may indicate high walkability of a county or it may be that these are counties with more road traffic and limited sidewalks. Unfortunately, given the resolution of the data and the need to cover the entire United States, it was not possible to untangle subtler meanings. This should be taken into account when interpreting the observed associations that are inverse or negative.

Strengths of this analysis include the use of the broad environmental context that covers hundreds of exposures simultaneously across several media and the ability to examine effects across the entire United States. Much of environmental research focuses on single exposure models without other environmental factors, or adjusts for potentially highly correlated environmental factors which makes interpretation of effect estimates more difficult. We used birth registry data for the entire United States in this analysis, enabling a large, population-based study. Confounding was unlikely given the comprehensiveness and “upstream” nature of the exposure and the inclusion of a proxy for race, though the possibility for effect measure modification, such as by race/ethnicity, remains.

## Conclusion

The EQI has the potential to be a useful tool in epidemiologic studies of PTB. Our study shows utility of the EQI at examining environmental associations with both county-level prevalences and individual levels odds of adverse health outcomes. Further research using the EQI and its constituent domains could include them for adjustment when examining individual environmental exposures (e.g., adjusting for general air quality or the rest of the ambient environment when examining the effects of particulate matter in detail). Although the research presented here is not without limitations, we believe the EQI to be a useful environmental quality metric at the county level and to have meaningful potential for future use.

This study was a cross-sectional analysis of PTB and environmental quality, using a new metric of composite environmental quality. We observed both positive and negative associations for preterm birth with overall environmental quality and with domain-specific indices in both county and individual analyses. We observed different patterns of effect across urban–rural strata, particularly for the sociodemographic domain, and to a lesser extent the air domain, potentially highlighting the relative importance of different domains depending on the degree of urbanicity. This study takes the focus from single harmful environmental exposures to a broader view of the environment encompassing several domains, and provides context for further studies of birth outcomes and the broad environment.

## Abbreviations

CI: Confidence interval
EPA: United States Environmental Protection Agency
EQI: Environmental Quality Index
NCHS: National Center for Health Statistics
OR: Odds ratio
PCA: Principal component analysis
PD: Prevalence difference
PTB: Preterm birth
RUCC: Rural–urban continuum code
US: United States
